# Generation of retinal pigmented epithelium from iPSCs derived from the conjunctiva of donors with and without age related macular degeneration

**DOI:** 10.1371/journal.pone.0173575

**Published:** 2017-03-10

**Authors:** Zhouhui Geng, Patrick J. Walsh, Vincent Truong, Caitlin Hill, Mara Ebeling, Rebecca J. Kapphahn, Sandra R. Montezuma, Ching Yuan, Heidi Roehrich, Deborah A. Ferrington, James R. Dutton

**Affiliations:** 1 Stem Cell Institute, University of Minnesota, Minneapolis, Minnesota, United States of America; 2 Department of Ophthalmology and Visual Neurosciences, University of Minnesota, Minneapolis, Minnesota, United States of America; 3 Histology Core for Vision Research, University of Minnesota, Minneapolis, Minneapolis, Minnesota, United States of America; University of Florida, UNITED STATES

## Abstract

Fidelity in pluripotent stem cell differentiation protocols is necessary for the therapeutic and commercial use of cells derived from embryonic and induced pluripotent stem cells. Recent advances in stem cell technology, especially the widespread availability of a range of chemically defined media, substrates and differentiation components, now allow the design and implementation of fully defined derivation and differentiation protocols intended for replication across multiple research and manufacturing locations. In this report we present an application of these criteria to the generation of retinal pigmented epithelium from iPSCs derived from the conjunctiva of donors with and without age related macular degeneration. Primary conjunctival cells from human donors aged 70–85 years were reprogrammed to derive multiple iPSC lines that were differentiated into functional RPE using a rapid and defined differentiation protocol. The combination of defined iPSC derivation and culture with a defined RPE differentiation protocol, reproducibly generated functional RPE from each donor without requiring protocol adjustments for each individual. This successful validation of a standardized, iPSC derivation and RPE differentiation process demonstrates a practical approach for applications requiring the cost-effective generation of RPE from multiple individuals such as drug testing, population studies or for therapies requiring patient-specific RPE derivations. In addition, conjunctival cells are identified as a practical source of somatic cells for deriving iPSCs from elderly individuals.

## Introduction

Research and clinical treatments using minimally manipulated cells isolated directly from donor tissue have the advantage that the primary cells may faithfully recapitulate their original function. However, these studies are often limited by restricted access to donor tissue, insufficient cell numbers and ethical considerations. In contrast, controlling the differentiation of pluripotent stem cells, either embryonic stem cells (ESCs) or induced pluripotent stem cells (iPSCs), can remove limitations on the scale of the manufacture of desired cell populations. However, efficient translation requires the generation of cell phenotypes from pluripotent stem cell intermediates with sufficient yield, purity and function to extend studies beyond proof of principle and lack of reproducibility in directed differentiation protocols remains a potential hurdle to improving the utility of these cell products. None the less, early stage clinical application of cells derived from ESC or iPSC intermediates is now being achieved following extensive optimization of cell differentiation and manufacturing processes [1–6].

Retinal pigmented epithelium (RPE) is one cell type that has been derived from pluripotent stem cells with sufficient efficiency and function to enter the early stages of clinical translation. In the eye, the RPE comprises a single layer of post-mitotic cells in intimate contact with the photoreceptors [7]. The RPE layer has a multitude of physiological roles including light absorption, phagocytosis, solute transport and growth factor secretion and maintenance of RPE function is critical for photoreceptor survival and function throughout the life of an individual. In conditions of macular degeneration, progressive loss of RPE is associated with increasing loss of vision and therapies to replace damaged RPE with RPE derived from ESCs are showing initial promise in early stage clinical trials [5, 8, 9]. In addition, the Riken Center for Developmental Biology in Japan has reported no initial safety concerns from the world’s first autologous iPSC-derived RPE transplant carried out in 2014.

The initial protocols for differentiating RPE from ESCs relied on manual isolation of regions of pigmented cells from cultures differentiated in the presence of exogenous stromal cells [10], on spontaneously differentiating stem cell cultures [11] or on early attempts to recapitulate non-neural retinal development *in vitro* [12]. Recently published directed differentiation protocols demonstrate more efficient initial induction of RPE from ESCs and iPSCs in as little as 14 days [13–15], a time scale more compatible with the cost effective differentiation that is required for large scale population studies, autologous transplant manufacturing and comprehensive drug screening regimes. To fulfil their potential, these applications require a scalable method to generate multiple RPE lines without using integrating reprogramming methods or requiring protocol optimizations for every new iPSC line and individual donor or process modifications for different manufacturing sites or laboratories.

A key goal of this study was to validate a reproducible process to enable simplified, rapid and cost-effective generation and expansion of RPE from multiple individuals with and without age-related macular degeneration (AMD). While primary RPE cells cultured from the retinas of eyes donated for research provide an invaluable research resource, primary RPE derivations are limited by the availability and cost of donor eyes and proliferation restriction reduces the numbers of cells available for experiments. Primary RPE cultures cannot be established from living patients and the generation of RPE by differentiating iPSCs is currently the most promising route for producing RPE from patient populations. However, the derivation of RPE from pluripotent stem cells has been inefficient, costly and labor intensive.

In this study, we examined the outcome from combining a fully defined iPSC derivation method [16, 17] with a rapid directed differentiation technique for RPE generation [14, 18], to determine the feasibility of generating multiple RPE lines from elderly donors with AMD and from age-matched healthy donors, without protocol adjustment and optimization for each individual cell line or donor. We report the characterization of RPE generated from multiple iPSC lines derived from conjunctival cells cultured from donor eyes graded for AMD disease. We show that conjunctival cells provide a practical alternative to skin fibroblasts for deriving iPSC lines from an elderly donor population and that conjunctiva iPSC-derived RPE can be readily generated using a defined, highly reproducible process suitable for research and clinical projects where large numbers of individual RPE cell lines are required.

## Materials and methods

### Human tissue procurement and grading

De-identified donor eyes were obtained from the Minnesota Lions Eye Bank, which receives these samples from deceased individuals with the written consent of the donor or donor’s family for use in medical research in accordance with the Declaration of Helsinki. The Minnesota Lions Eye Bank is licensed by the Eye Bank Association of America (accreditation #0015204) and accredited by the FDA (FDA Established Identifier 3000718538). Donor tissue is exempt from the process of Institutional Review Board approval. Evaluation of the donor’s stage of AMD was determined by a Board Certified Ophthalmologist (SM) from stereoscopic fundus photographs of the RPE using the criteria established by the Minnesota Grading System (MGS) [19, 20]. MGS 1 represents the control group with no clinically observable eye disease. MGS 2, 3, 4 are early, intermediate, and advanced stages of AMD, respectively. The Minnesota Lions Eye Bank provided donor demographics.

### Primary conjunctival cell culture

Donated eyes were examined and samples with pannus, hemorrhage or any other abnormalities were not utilized. In a laminar flow hood, the endothelium and iris were removed from the eye using jewel scissors. From a single corneal button in a 10cm diameter tissue culture dish the corneal epithelia was separated from the sclera up to approximately 2mm from the limbus region. The separated conjunctival tissue then was dissected circumferentially and the tissue pieces placed in Dulbecco’s Phosphate buffered saline (DPBS) (ThermoFisher, Waltham, MA, USA. 14190144) and dissected into 0.5-1mm x0.5–1mm pieces using no. 21 scalpel blades. The conjunctival pieces were dispersed and allowed to partially air dry for 30 seconds before being covered with 30μl of keratinocyte growth medium, KGM-CD (Lonza, Basel, Switzerland. cc-4455) and connected to a 3ml reservoir of media before incubation overnight at 37°C, 5% CO_2_. KGM-CD media (1 ml) was added to the media reservoir every 2 days for 7 days. After 7 days, the medium was replaced with 5ml fresh KGM-CD medium, replaced every 2 days for another 7 days. The cells were then harvested using 0.2% Trypsin/EDTA neutralized with soybean trypsin inhibitor.

### Induced pluripotent stem cell reprogramming of human conjunctival cells

The derivation of iPSCs from conjunctival cells was conducted using the CytoTune^™^ 2.0 Sendai Reprogramming Kit (A16517, Thermo Fisher Scientific, Waltham, MA, USA) following the manufacturer’s instructions. Primary human conjunctival cells were cultured in KGM-CD medium at 5–10 x 10^4^ cells/cm in a 9.6cm^2^ plate. Cells at 70–80% confluence were then transduced with Sendai vectors as directed by the manufacturer and incubated overnight at 37°C, 5% CO_2_. The medium was replaced with fresh KGM-CD and cultured for 7 days with medium changes every other day. Seven days after transduction the cells were passaged onto culture dishes coated with 0.5μg/cm^2^ recombinant human vitronectin (AF14009, PeproTech, Rocky Hill, NJ.) at 5x10^4^ cells/cm^2^ and cultured in KGM-CD for 24 hours before changing to Essential 8 medium (A1517001, Thermo Fisher Scientific, Waltham, MA, USA.) with daily media changes at 37°C, 5% CO_2_. Emerging iPSC colonies were identified by morphology, picked manually and transferred to vitronectin coated 3.7cm^2^ wells. iPSC lines were then expanded using hypertonic citrate passaging essentially as described in [16, 17]. To characterize vector loss, RNA was extracted from 2 x 10^6^ cells using RNeasy Plus kits (Qiagen). cDNA synthesis was performed using Superscript III (ThermoFisher), and 1μg RNA-equivalent cDNA was used for quantitative reverse-transcription PCR (qRT-PCR) with custom PrimeTime Assays (Sendai vector-specific Ref. No. 148160468, Gapdh Ref No. 148160472, IDT Technologies) on an Eppendorf Mastercycler (Realplex^2^). qRT-PCR analysis was used to analyze the expression of selected pluripotent stem cell related genes using RNA extracted from the iPSC lines following expansion and banking. Immunocytochemistry detected Oct4, Nanog and Tra 1–81 expression. G-banded karyotyping was performed by Cell Line Genetics (Madison, Wisconsin). For teratoma analysis 2 x10^6^ cells were suspended in 50% v/v Matrigel (354277, Corning, Corning NY) and injected into the hind limb muscle of NOD-SCID mice (Jackson Laboratories). Mice were observed up to 12 weeks before teratoma recovery. Haematoxylin and eosin histology on paraffin sections was performed to observe gross morphology and the tumors were scored by a veterinary pathologist for evidence of complex teratoma formation containing tissues derived from the three embryonic germ layers. Animal experiments, including euthanasia by CO_2_ overdose, were carried out under protocol 1601-33304A approved by the University of Minnesota Institutional Animal Care and Use Committee.

### Initial directed differentiation of conjunctival iPSCs into Retinal Pigmented Epithelia (RPE)

**Fig 1:**
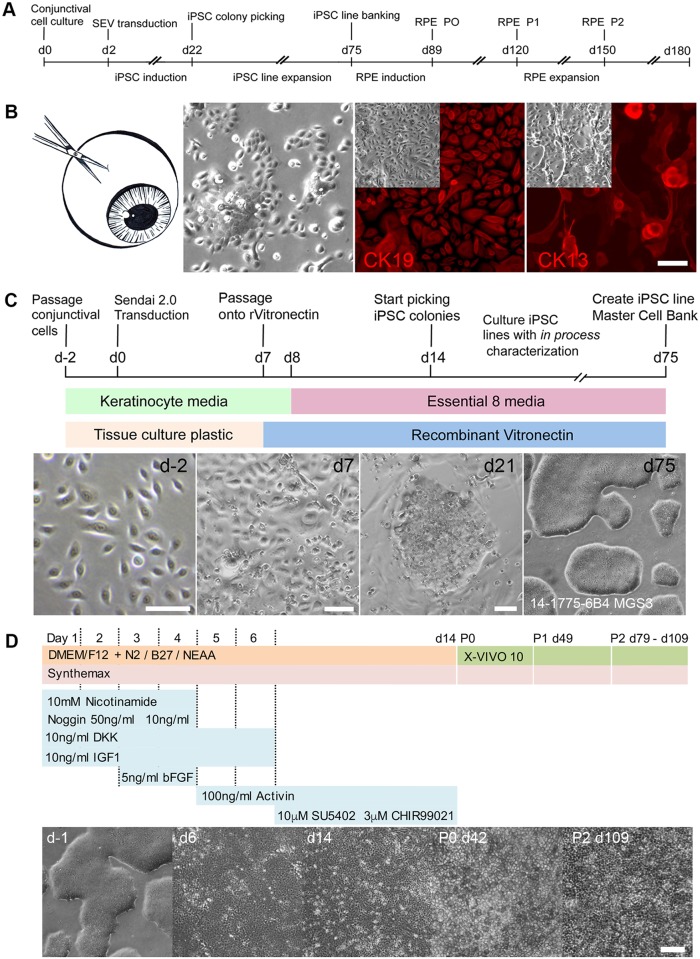
iPSC reprogramming and differentiation of conjunctival cells into RPE. **A** Timeline for iPSC derivation and RPE differentiation using cells isolated from human conjunctiva. **B** Conjunctival cells cultured from corneal epithelium biopsies generate adherent cell monolayers expressing CK19 and CK13 (Scale bar 50μm). **C** Protocol and timeline for reprogramming human conjunctival cells into iPSCs under fully defined conditions. iPSC colonies identified by characteristic morphology are expanded to derive individual iPSC lines for characterization and banking. **D** Schematic representation of the protocol for rapid differentiation and maintenance of RPE from conjunctival iPSC lines under fully defined conditions. Phase microscopy illustrating the appearance of the cell monolayers during the initial differentiation of iPSCs into RPE and during the maintenance and expansion of the iPSC-derived RPE. (Scale bar 100μm).

The protocol for differentiation of pluripotent hiPSCs into RPE was adapted from Buchholz et al., 2013 [14]. For differentiation, iPSCs were plated at 70–80% confluence on 9.6cm^2^ wells coated with 1X Matrigel (Corning) or Synthemax (3535XX1 Corning, Corning NY) or vitronectin (PeproTech) in basal neural induction medium (DMEM/F12+ 1X N2+ 1X B27 + 1X Non-essential amino acids, Thermo Fisher Scientific) supplemented for 2 days with Nicotinamide (10mM) (Sigma-Aldrich, St. Louis, MO), Noggin (50ng/ml) (PeproTech), Dkk-1 (10ng/ml) (R&D Systems, Minneapolis, MN.) and IGF-1 (10ng/ml) (R&D Systems) with a medium change on day 1. On days 3 and day 4, the Noggin concentration was reduced to 10ng/ml and bFGF was added at 5ng/ml (R&D Systems). The Nicotinamide, bFGF and Noggin were removed from the media on day 5 and on days 5 and 6 the basal medium was supplemented with only Dkk-1 (10ng/ml), IGF1 (10ng/ml) and Activin A (100ng/ml) (PeproTech). The DKK and IGF 1 were then removed and from day 7 to day 14 the basal medium was supplemented with Activin A (100ng/ml), and SU5402 (10μm) (Sigma-Aldrich). CHIR 99021(3μM) (TOCRIS, Bristol, UK.) was also added from day 8 to day14. (See Fig 1). All media supplements were removed for the expansion of iPSC-derived RPE.

## Enrichment and expansion of iPSC-derived RPE cells

On day 14 the cells were passaged with Accumax (A7089, Sigma-Aldrich) at a density of 1x10^5^/cm^2^ in XVIVO-10 (04-743Q, Lonza, Basel, Swizerland) on 1X Matrigel, Synthemax or vitronectin coated dishes, with ROCK inhibitor Y27632 (10μM) (S1049, Selleckchem, Houston, TX.) for 7 days with media changes on day +1 and +5. These cells were termed Passage 0. After 7 days the Y27632 was removed and P0 cells were cultured in XVIVO 10 medium for 35 days with 2 medium changes per week. Cells were dissociated with Accumax and passaged at 1x10^5^ cells/cm^2^. From P1 to P5, RPE cells were cultured for 30 days in each passage.

### RNA isolation and qPCR assay of RPE gene expression

Total RNA was prepared with the RNeasy Micro kit (Qiagen, Valencia, CA). 150ng/μl RNA was used to synthesize cDNA (SuperScript III First-strand Synthesis System, ThermoFisher Waltham, MA, USA 18080–051). Quantitative PCR analysis was performed using SYBR Green (13962800, Roche, Indianapolis, IN) with an Eppendorf Mastercycler (Realplex^2^). Amplification primers are listed in Table 1. Relative gene expression was calculated by the ΔΔCT method. GAPDH gene expression was used to normalize expression data and gene expression at each time point is indicated relative to undifferentiated iPS cells.

**Table 1:** Primers for gene expression analysis used in this study.

Gene		Sequence (5'->3')	Amplicon (bp)	Tm	NCBI Gene ID
MITF	F	CCCAGTTCATGCAACAGAGAG	79	58.9	4286
	R	GCAGAGGGAAGGGTGGTG		59.7	
RPE65	F	CGTATGGACTTGGCTTGAATC	129	57.1	6121
	R	CTGGGTGAGAAACAAAGATGG		56.8	
PMEL17	F	TGATGGCTGTGGTCCTTGC	94	60.3	6490
	R	CAGTGACTGCTGCTATGTGG		58.6	
PEDF	F	TATCACCTTAACCAGCCTTTCA	83	57.1	5176
	R	GGGTCCAGAATCTTGCCAATG		59.3	
TYRP1	F	GATTCCACTCTAATAAGCCCAAA	198	56.3	7306
	R	TTCCAAGCACTGAGCGACAT		60.0	
BEST1	F	TAGAACCATCAGCGCCGTC	261	60.0	7439
	R	TGAGTGTAGTGTGTATGTTGG		55.3	
RBP1	F	CAGGTTGGGAAGGAGTTTGA	168	57.3	5947
	R	TGGTGGCGTGTGACTGTAAT		59.6	
PAX6	F	TGTGTGCTCTGAAGGTCAGG	170	59.6	5080
	R	CTGGAGCTCTGTTTGGAAGG		58.2	
GAPDH	F	GAGTCAACGGATTTGGTCGT	185	58.2	2597
	R	GACAAGCTTCCCGTTCTCAG		58.6	
GAPDH	F	GTGGACCTGACCTGCCGTCT	153	61.7	2597
	R	GGAGGAGTGGGTGTCGCTGT		61.7	
OCT4	F	CCTCACTTCACTGCACTGTA	164	54.3	5460
	R	CAGGTTTTCTTTCCCTAGCT		52.2	
NANOG	F	CAGAAGGCCTCCGCACCTAC	111	60.2	79923
	R	ATTGTTCCAGGTCTGGTTGC		55.6	
SOX2	F	GGGAAATGGGAGGGGTGCAAAAGAGG	151	63.6	6657
	R	TTGCGTGAGTGTGGATGGGATTGGTG		63.1	
LIN28	F	ACCGGACCTGGTGGAGTATT	133	58.0	79727
	R	CTGGGGATGCCTTAGCAAC		58.4	
HDNMT3B	F	CAAATGAACCCTGCGCTGTC	207	57.1	1783
	R	CAGGTCCCCAGATTCCCTCA		58.6	
REXO1	F	CCCACGTGCGCTGATCTCT	134	60.1	57455
	R	CTGAGGTAGTCGGCCATGAG		57.1	
TERT	F	CTCAGGACAGGCAAGTGTGG	232	58.1	7015
	R	ATGGGAAGCAGGGGTTCAAG		57.6	

### Immunofluorescence and microscopy

Cells were fixed with 10% buffered formalin (305–510, Protocol, Kalamazoo, MI) at room temperature for 10 minutes, washed with DPBS without Ca and Mg (14190144, ThermoFisher) and stored at 4°C. Cells were permeabilized with 0.2% Triton X-100 (Sigma) and blocked with blocking buffer (1% BSA (820451, MP Biomedicals) + 0.1%Triton X-100 +10% serum matched to the hosts of secondary antibodies) for 30 minutes. Cells were then incubated overnight at 4°C with primary antibodies (see Table 2) diluted in blocking buffer. Cells were washed three times with PBS-T (PBS without Ca and Mg + 0.1% triton x100 + 1% BSA) before incubating with secondary antibodies diluted in blocking buffer for at least 1 hour at room temperature. Cells were then washed three times for 5 minutes with PBS-T and DAPI (Roche) was added. Histology and immunohistochemistry was analyzed using a Zeiss Axiovert 200M inverted microscope fitted with an AxioCam camera. For confocal microscopy the cells were cultured on transwell inserts and imaging was obtained using an Olympus FluoView 1000m IX81 inverted confocal microscope. Z-stack images were analyzed using ImageJ software (NIH, USA) and Imaris (Bitplane, Oxford Instruments).

**Table 2:** Antibodies used in this study.

Antigen	Host	Company	Product	Dilution
TRA-18-1	Mouse	Millipore	MAB4381	1:100
NANOG	Goat	R&D Systems	AF1997	1:100
OCT4	mClone 10H11.2	Millipore	MAB4401	1:250
PAX6	Mouse	DHSB	Pax6	1:25
MITF	Rabbit	Abcam	ab122982	1:500
BEST1	Mouse	Novus	NB300-164	1:200
OTX2	Rabbit	Millipore	AB9566	1:500
RPE65	Mouse	Novus	NB100-355	1:200
PMEL17	Mouse	Dako	M063429-2	1:200
ZO-1	Rabbit	Invitrogen	61–7300	1:500
CHX-10	Rabbit	Novus	NBP184476	1:200
Confocal Microscopy				
ZO-1	Mouse	Invitrogen	339100	1:200
BEST	Rabbit	Abcam	Ab14927	1:100
Na^+^/K^+^ATPase	Mouse	Novus	NB300-146	1:100
EZRIN	Mouse	Cell Signaling	3145	1:200

### ELISA

RPE cells were seeded at a density of 2x10^5^ cells/well in 6.5mm diameter polyester inserts (0.4μm pores; Corning, Inc.) coated with 1X Matrigel (Corning). Cell monolayers were cultured for approximately 2 months with media changes twice per week. Medium was collected from both apical and basal chambers following 24-hour exposure of fresh media to the cells. ELISA for vascular endothelial growth factor A (VEGF A; Life Technologies) and pigment epithelium-derived factor (PEDF; R & D Systems) were conducted according to the manufacturer’s protocols. Growth factor concentrations were derived from standard curves and corrected for chamber volume differences.

### Rod outer segment phagocytosis

Bovine rod outer segments were obtained from InVision BioResources (Seattle, WA). The outer segments were labeled with FITC (Fluorescein-5-Isothiocyanate Isomer I) (Molecular Probes) as described previously [21]. Briefly, FITC was resuspended to a final concentration of 2.5 mg/mL in 0.1M sodium carbonate buffer pH 9.5, by mixing for 1 hour at room temperature while protecting from light. The ROS pellet containing approximately 200 million OS was resuspended in 3.125 mL ROS Buffer (10% sucrose, 20mM Sodium Phosphate pH 7.2, 5mM Taurine) and incubated for 1.5 hour at room temperature with 0.625 mL FITC solution while rotating and protecting from light. Labeled OS were pelleted at 3000xg for 10min and then washed two times in ROS buffer. A final wash was done in XVIVO-10 media. ROS were resuspended in XVIVO-10 and added to confluent, adherent RPE at a concentration of 40 ROS /cell. The cells were incubated for 16 hours at 37°C, 5% CO_2_. RPE cells were then washed 3 times with PBS without Ca and Mg. 1X Accumax (Sigma) was applied for cell dissociation and single cells were collected, washed with PBS without Ca and Mg and resuspended in PBS with 1% BSA. Flow cytometry analysis was performed using a BD LSRFortessa (BD Biosciences) and the data analyzed with FlowJo software.

## Results

### Conjunctival cells for iPSC reprogramming and RPE differentiation

A timeline for reprogramming primary conjunctival cells into iPSCs and their differentiation into RPE is shown in Fig 1A. In this study, the conjunctival tissue was obtained from eyes donated to the Minnesota Lions Eye bank. Retinal images from each donated eye allowed grading for the extent of AMD disease using the Minnesota Grading System (MGS) [20]. Primary cultures of conjunctival cells were obtained from explants dissected from the corneal epithelial tissue covering the sclera. The tissue explants were cultured in defined keratinocyte media to generate a population of proliferative epithelial cells expressing Cytokeratin 19 and Cytokeratin 13 (Fig 1B). To model clinical conjunctiva tissue biopsy procedures, 2mm punch biopsies were collected in a subgroup of donors, from the superior, inferior, nasal and temporal conjunctival regions from each donor eye and placed in culture as described. After 2 weeks in culture, an average of 3.52 x10^5^ cells / 2mm biopsy was obtained (n = 3 donors). (S1 Fig). The cell yield per biopsy was sufficient to provide cells for reprogramming, expansion and banking.

### Reprogramming conjunctival cells with non-integrating Sendai virus vectors

Differentiation of iPSCs into defined cell types is heavily influenced by the methods and materials employed for somatic cell reprogramming and subsequent iPSC culture and differentiation. To enhance the reproducibility of our protocols we have implemented a fully defined procedure for somatic cell reprogramming and iPSC expansion that is amenable to both research and clinical cell manufacturing procedures [16, 17] (Fig 1C) and intended to ensure that iPSC lines derived and differentiated for research studies are generated in the same manner as proposed for potential future translation. Conjunctival cells were reprogrammed using CytoTune^®^-iPS 2.0 non integrating Sendai virus based vectors (Fig 1C). Colonies with characteristic iPSC morphology were evident 14 days after transduction under fully defined culture conditions and individual colonies were picked manually, beginning 21 days after transduction and expanded to derive multiple iPSC lines from each donor. Details of the donors and iPSC lines used in this study are shown in Table 3. Our iPSC derivation system allows in-process testing of each line before generating master cell banks. All lines were examined prior to banking for maintenance of typical iPSC colony morphology and growth dynamics, expression of antigens associated with pluripotency and to ensure loss of exogenous reprogramming vectors. Following expansion and banking each line was also characterized to ensure a normal karyotype by G banding analysis, characteristic pluripotent stem cell associated gene expression and the ability to generate a complex teratoma when differentiated *in vivo* in immune deficient hosts (S2 Fig). Successful establishment of conjunctival cell cultures from donors aged 70–85 and the derivation of multiple iPSC lines from each donor illustrates the utility of using primary conjunctival cell cultures as a practical cell source for iPSC reprogramming from elderly individuals.

**Table 3:** Donor characteristics for the iPSC lines derived in this study.

Donor ID	Sex, Age	Graded AMD	UMN iPSC Line ID
UMN-AMD1^a^	Male, 71yr	MGS1	UMN AMD1-1D3
			UMN AMD1-2B6
UMN-AMD1.1^b^	Female, 77yr	MGS1	UMN AMD1-2C3
			UMN AMD1-3A3
UMN-AMD2^c^	Male, 76yr	MGS2	UMN AMD2-1A1
			UMN AMD2-1C1
			UMN AMD2-2A4
UMN-AMD3^d^	Female, 85yr	MGS3	UMN AMD3-3C1
			UMN AMD3-6B4
			UMN AMD3-2B1
UMN-AMD3.1^e^	Male, 79yr	MGS3	UMN AMD3-1B1
			UMN AMD3-1C4

Whole eyes were donated for research to the Minnesota Lions Eye Bank, Minneapolis MN. All donors were Caucasian. Cause of death:

^a^ acute cardiac arrest,

^b^ anoxic brain injury,

^c^ sepsis,

^d^ breast cancer,

^e^ stroke.

### Differentiation of conjunctival-derived iPSCs into Retinal Pigmented Epithelium (RPE)

Manual isolation of pigmented cell patches generated following spontaneous differentiation of hESCs has been used extensively to generate RPE for research and clinical use [5, 11, 12, 22]. However, differentiation protocols incorporating undefined and xenobiotic reagents, are unpredictable and require significant manual interventions that are undesirable in manufacturing processes and when multiple cell lines are differentiated in parallel. More defined protocols have now been described improving the speed, yield and purity of RPE differentiation from human ESCs and IPSCs. In this work we have adopted protocols from the Clegg Laboratory [14, 18] to reproducibly generate high yields of RPE from multiple iPSC lines without the requirement for manual enrichment (Fig 1D). The protocol induces a RPE phenotype in adherent iPSC cultures in 14 days. After d14 the early RPE cells are passaged and the iPSC-derived RPE cells are expanded every 30 days to generate large numbers of cells for experiments that may require significant RPE cell populations.

**Fig 2:**
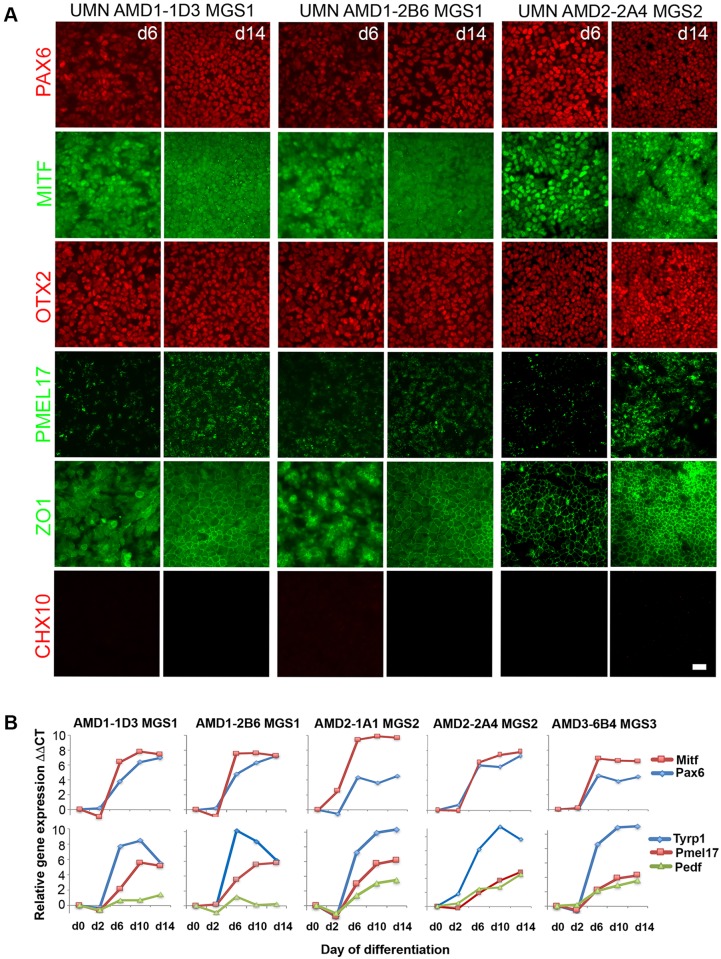
Characterization of the initial induction of RPE from donors with and without AMD. iPSC lines derived from donors with and without AMD were differentiated into RPE using the rapid 14 day induction protocol. **A** Immunohistochemistry detected the expression of proteins indicating the rapid establishment of early eye field and RPE identity at 6 and 14 days after the start of directed differentiation. Protein expression is shown from the differentiation of 2 iPSC lines from the same donor (UMN AMD1-1D3, UMN AMD1-2B6 –MGS1 no disease) and from iPSC line UMN-AMD2-2A4 (MGS2—AMD disease). (Scale bar 50μm) (B) qRT-PCR analysis comparing the time course of eye field and early RPE gene expression during RPE differentiation in multiple iPSC lines derived from three donors with and without AMD.

Monitoring the rapid induction of RPE identity in multiple iPSC lines is described in Fig 2. Immunohistochemistry from the directed differentiation of RPE cell lines derived from the same donor (UMN AMD1-1D3, UMN-AMD1-2B6, 71 year old male—MGS1 no AMD) and a line derived from a donor with early AMD disease (UMN AMD2-1A1, 74 year old male, MGS2) are shown in Fig 2A. Quantitative gene expression analysis for these lines together with a second line from the MGS2 donor (UMN AMD-2A4) and a line derived from a donor with more advanced AMD (UMN AMD3-6B4, 85 year old female, MGS3) are shown in Fig 2B. Markers of early eye field identity are upregulated rapidly in the presence of the retinal inducing factors Noggin, DKK and IGF1. Pax6 and Mitf gene expression was induced by d6 with corresponding nuclear protein localization that is maintained d14 post-induction. The expression of the pigmentation genes pre-melanosome protein 1 (Pmel17) and tyrosinase related protein 1 (Tyrp1) were also induced by d10 with Pmel17 protein expression detected by d14 in each cell line. Evidence of the progression to RPE identity was shown by the formation of a homogeneous cell monolayer with resolution of the expression pattern of the tight junction protein ZO1. The addition of the selective glycogen synthase kinase 3 (GSK-3) inhibitor CHIR99021 has previously been shown to reduce the induction of non-RPE contaminating cell types in this differentiation process [18] and in our experiments neuronal cells expressing CHX10 were largely undetectable 14 days after induction. The similarity of gene and protein induction patterns displayed during the RPE differentiations using iPSC lines from the the same donor or different donors, demonstrates that the combination of our defined iPSC culture system and this protocol allows RPE to be produced efficiently and reproducibly from multiple donors without requiring prohibitive optimization for individual cell lines, an essential requirement for both population studies and cell manufacturing paradigms.

### Expansion and maintenance of iPSC-derived RPE cultures

**Fig 3:**
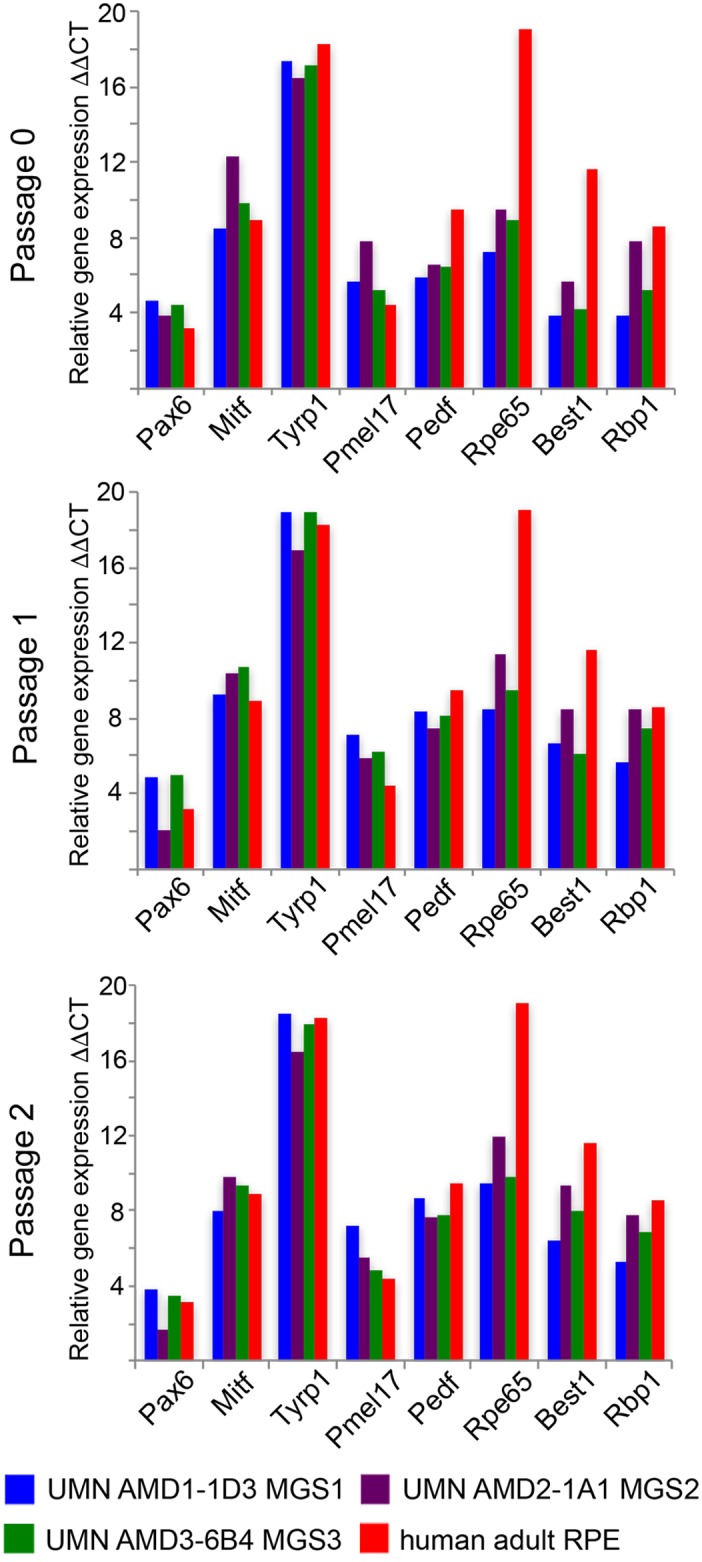
Gene expression in iPSC-derived RPE cell lines generated from donors with and without AMD. iPSC lines derived from donors with and without AMD were differentiated into RPE and the RPE maintained and expanded over three passages (PO-P2). Expression of RPE related genes was analyzed for each line and shown in comparison with the expression level in human adult RPE isolated directly from an age matched control donor without AMD.

Conjunctival iPSC-derived RPE cells from each donor were passaged for expansion and maintenance. Homogeneity in the initial RPE induction enabled passaging without the necessity for manual enrichment of sporadic pigmented regions, significantly reducing process complexity at the initial RPE culture expansion stage. To monitor the maintenance of RPE identity, quantitative gene expression of selected RPE markers was analyzed over consecutive passages for iPSC-derived RPE lines generated from three donors with different levels of diagnosed AMD disease (Fig 3). The gene expression level was compared with that in mRNA (human adult RPE) isolated directly from the RPE of an age-matched donor (87 year old male) without AMD disease. The mRNA expression levels of Pax6, Mitf, Tyrp1, Pmel7, PedF and Rbp1 in the iPSC-derived RPE lines tested were similar to the expression level detected in uncultured adult RPE and were maintained in all three lines tested during expansion from P0 to P2. Consistent gene expression of these markers of RPE identity was also displayed during expansion over 5 passages of two iPSC lines derived from the same donor, further demonstrating the reproducibility of the RPE induction and the expansion process (S3 Fig). The gene expression patterns of Best1, encoding a transmembrane calcium activated anion channel protein and Rpe65, encoding a 65kDa RPE associated protein, were consistent between the RPE cell lines generated from iPSCs maintained in culture. By Passage 2 the Best1 expression level matched that seen in adult RPE. However, the expression level of Rpe65 was consistently reduced compared with that detected in RPE dissected from donor tissue suggesting a possible requirement for interaction with photoreceptors to generate the level of gene expression found in the retina *in vivo*.

**Fig 4:**
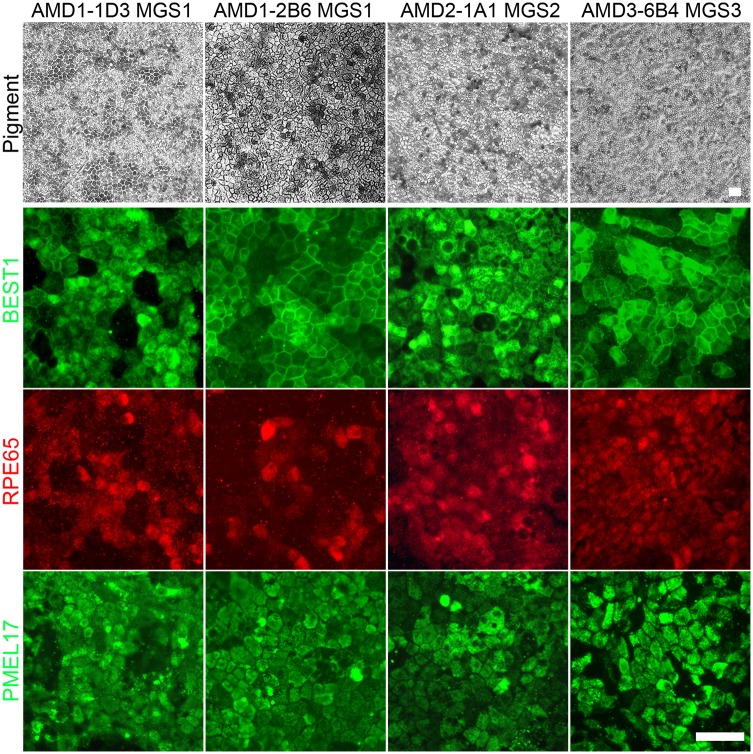
Immunohistochemistry of iPSC-derived RPE cell lines from donors with and without AMD. RPE cell lines generated from iPSC lines derived from donors with and without AMD were expanded in culture. Phase microscopy images were taken to illustrate the pigmented monolayer. Immunohistochemistry and fluorescent microcopy was used to detect the expression pattern of characteristic protein markers of RPE, the ion channel BESTROPHIN, 65kDa Retinal Pigment Epithelium-Specific Protein RPE65 and the pre-melanosome protein PMEL17. (Scale bar 50 μm).

Pigmentation and the protein expression pattern of BESTROPHIN, RPE65 and PMEL17 were also examined in expanded iPSC-RPE cultures (Fig 4). Each line exhibited a characteristic epithelial monolayer phenotype with dark pigmentation. Appropriate protein localization was observed for BESTROPHIN, PMEL17 and RPE65. The heterogeneous pattern of RPE65 expression is consistent with an additional maturation requirement for photoreceptor contact to enhance the expression of components of the visual cycle.

### Functional analysis of RPE derived from conjunctival iPSC lines

**Fig 5:**
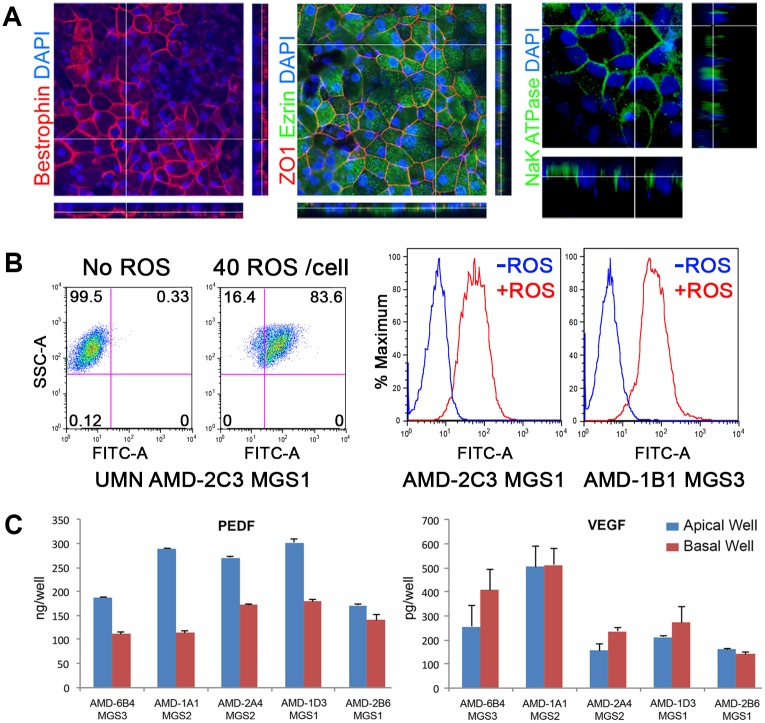
Functional analysis of polarized iPSC-derived RPE. Conjunctival iPSC-derived RPE cultured on transwell inserts before examination of monolayer polarity by immunohistochemistry and confocal microscopy. **A** *en face* views of the RPE monolayer shown as maximum intensity projections through the *z*-axis. Cross-sections through the *z*-plane of multiple optical slices, locations shown by the white line. Nuclei are stained using DAPI. **B** Phagocytosis of FITC-labelled bovine rod outer segments (ROS) by conjunctival iPSC-derived RPE cell lines. FACs analysis of iPSC-derived RPE from a donor with AMD and a healthy control donor with no AMD. Histograms illustrate the rightward shift for cells that have phagocytozed FITC labelled ROS. **C** Growth factors secretion by iPSC derived RPE. PEDF and VEGF secretion was measured for multiple iPSC-derived RPE cell lines. RPE cells were cultured in transwell inserts and polarized growth factor secretion in the apical or basal reservoirs was measured by ELISA.

Mature retinal RPE *in vivo* exhibits a polarized localization of specific proteins on the apical side facing the photoreceptors or the basal side adjacent to Bruch’s membrane. The *in vivo* condition can be partially replicated by growing cells on permeable transwell inserts. Confocal microscopy was used to examine the presence of stereotypical apical and basal protein expression patterns in the iPSC-derived RPE line UMN AMD2-1A1 MGS2 (Fig 5A). Bestrophin, Ezrin and Na-K ATPase protein expression was detected in the *en face* images and examination of the cross section indicated basolateral localization of Bestrophin, which is consistent with the protein in mature RPE. Z-stack analysis also indicated apical localization of both Ezrin, an actin-binding protein associated with apical microvilli and RPE-specific apical localization of Na-K ATPase. We also evaluated the ability of our conjunctival iPSC derived RPE to phagocytose fluorescent labelled bovine rod outer segments, an *in vitro* assay for the RPE’s function in recycling shed rod outer segments in the retina. FACs gating and histogram summaries of fluorescent cell sorting demonstrate ability to internalize FITC labelled rod outer segments by iPSC lines derived from different donors (Fig 5B). A further marker of mature functional RPE is the production of the angiogenic growth factor vascular endothelial growth factor (VEGF) and the anti-angiogenic growth factor pigment epithelium-derived factor (PEDF). Growth factor expression was measured by ELISA for iPSC-derived RPE cell lines derived from the same or different donors cultured in transwell inserts (Fig 5C). PEDF was predominantly secreted on the apical side (mean average expression (+/-SD) 243.8 ng/well (+/-2.8) apical, 143.2 ng/well (+/-4.8) basal) and total PEDF expression in the apical and basal wells was >900 fold and >450 fold than VEGF respectively. Polarized PEDF expression in the cultured RPE cells is consistent with *in vivo* PEDF expression in the eye [23] and indicates maintenance of correct polarization in the expanded iPSC-derived RPE. The results shown here are representative of those obtained for iPSC-RPE derived from all the eye bank donors tested in this study.

### GMP-compliant derivation of RPE from conjunctival iPSC lines

**Fig 6:**
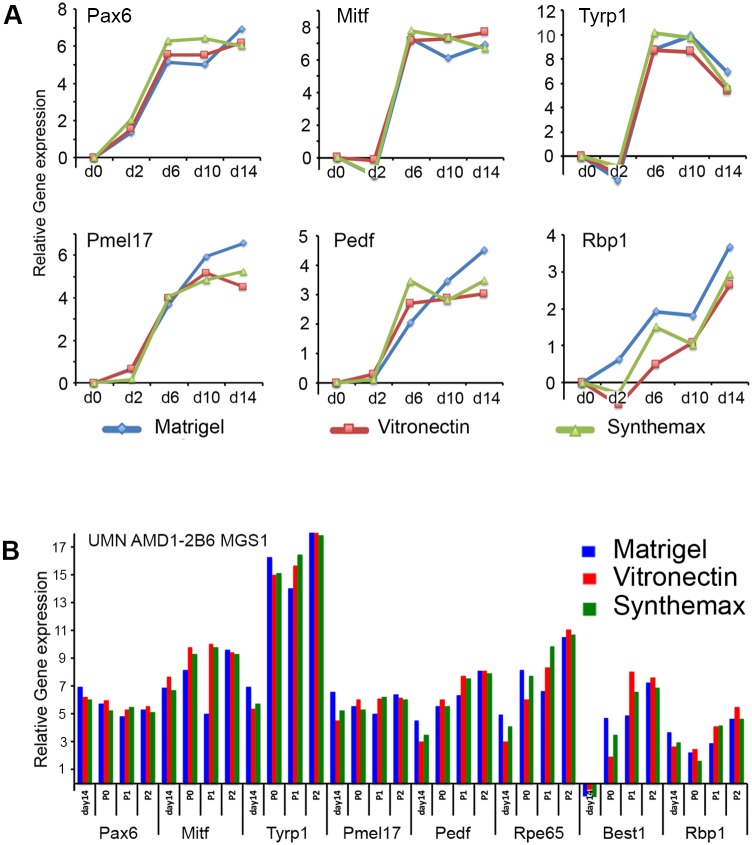
Derivation of RPE from conjunctival iPSCs using fully defined culture conditions. **A** Comparison of qRT-PCR analysis of key markers of initial RPE phenotype induction from iPSC line UMN AMD1-2B6 differentiated on three different substrates, undefined *Matrigel* or defined substrates vitronectin or *Synthemax*) compatible with requirements for cGMP-compliant RPE cell manufacture. Following the initial induction iPSC-derived RPE cultures were maintained and expanded on the different substrates for three passages. **B** Comparison of qRT PCR analysis of gene expression of RPE made from iPSC line UMN AMD1-2B6 maintained on the three different substrates.

The incorporation of chemically defined media and culture substrates in the design and validation of cell culture and differentiation protocols is critical to expanding the utility of published methods beyond individual research projects. The proposed use of iPSC-derived cell products for clinical transplants also requires the absence of xenobiotic and undefined reagents in cell and tissue manufacturing. To this end, we tested our RPE induction and expansion process using two defined substrates to substitute for the commonly used mouse sarcoma cell-derived substrate Matrigel^™^ (Fig 6). Synthemax (Corning) and recombinant vitronectin (PeproTech) are chemically defined matrices suitable for scalable coating of cell culture surfaces compatible with the current good cell and tissue manufacturing practices required in clinical cell production. Monitoring the gene expression of key RPE markers during RPE differentiation and expansion of conjunctival iPSC line UMN AMD1-2B6 (MGS1) indicated that progression of RPE induction and maintenance of RPE phenotype was not influenced by the culture substrate and that the substitution of the undefined Matrigel substrate with either one of the defined matrices resulted in successful preparation of RPE.

## Discussion

Generating authentic retinal pigmented epithelial cells from human pluripotent stem cells is a critical step for many current research and clinical projects. The ability to make large numbers of functional RPE from ESCs or iPSCs eliminates restrictions imposed by the limited availability and proliferation constraints of using primary human RPE and has introduced new research and therapeutic opportunities. These include the ability to generate RPE from individual donors with and without macula disease, from populations with known disease risk factors and the ability to generate RPE suitable for clinical cell replacement therapies. However, current paradigms for generating specific cell types by directing the differentiation of undifferentiated stem cells often utilize complex protocols designed to mimic embryonic development with successive restriction of cell identity promoted by the manipulation of *in vitro* culture conditions with timed application of growth factors and cytokines. Although these protocols may generate specific cell types with adequate function and yield for proof of principle research, the results may be difficult to reproduce in different laboratories or with different pluripotent stem cell lines. Lack of precision in protocol design and implementation, diversity in cell lines and culture techniques and the use of undefined reagents can contribute to this issue [24–26]. Published protocols may also have been optimized to promote differentiation of specific pluripotent stem cell lines and extending their use for new cell lines in different settings can involve extensive revision of individual protocol steps.

Ideally, standardization of pluripotent stem cell culture and differentiation methods will produce protocols with predictable outcomes that could be implemented successfully with any starting cell line without requiring additional optimization. Progress in the commercial manufacture and distribution of a range of chemically defined culture media systems and substrates now permits the establishment of standard methods for pluripotent stem cell culture so that undifferentiated cells entering differentiation protocols can exhibit similar properties in any research or manufacturing location. The exclusive use of non-integrating reprogramming methods, chemically defined media, additives and matrices should be routine in the design of reprogramming and directed differentiation protocols to enhance the precision and reproducibility of the method whether targeted at research or cell manufacturing for clinical use.

In this study we have applied these principles to the differentiation of retinal pigmented epithelium (RPE) from iPSC lines derived from multiple donors. Our research group is interested in studying the changes in RPE physiology that occur in the retina of individuals who experience AMD and finding new ways to prevent the progression of this devastating condition of aging. Critical insights into the progression and effect of this disease on the RPE have been obtained using primary RPE from eyes donated post mortem [19, 27, 28]. However, we wish to utilize the ability to generate RPE from iPSCs to extend this research to include living patients, to investigate new ways to prevent RPE damage in AMD and to study the feasibility of autologous RPE manufacture for potential transplant in patients progressing towards vision loss due to RPE cell loss.

The introduction of human conjunctival epithelial cells for iPSC derivation in this project enables the use of this same cell source for reprogramming from both living patients and post mortem eye tissue. The ability to generate iPSCs from conjuctival cells has previously been demonstrated in mice [29]. Standardizing the cell type for reprogramming is designed to reduce one source of variation in reprogramming outcomes described when comparing iPSC lines derived using cell types of different origin [30]. Eyes donated to the Minnesota Lions Eye bank are graded for AMD disease and provide access to tissue from individuals with all stages of AMD disease as wells as age matched healthy controls. Our work demonstrated the ease of establishing conjunctival cell cultures from multiple individuals of advanced ages providing a new practical source of cells for iPSC reprogramming that can be readily translated to AMD clinics as conjunctival biopsies are well tolerated by patients. The consistency of iPSC line derivation from conjunctival cells isolated from eye bank donors aged 70–85 indicates that this cell type may not exhibit the age related decline in reprogramming efficiency previously found using dermal fibroblasts [31]. This is an important consideration in AMD research where all of the patients are elderly. The use of conjunctival cells with defined *Essential 8* media, recombinant vitronectin substrate and commercially available non integrating Sendai virus based vectors together with simple chelation passaging produces a standardized method of iPSC reprogramming that can be readily transferred to other groups.

Generating RPE cells from iPSC lines derived from multiple donors requires a directed differentiation protocol that generates consistent yields of functional RPE from each line at a scale, speed and cost that does not compromise experimental planning. Recently published RPE differentiation protocols have markedly increased the speed and efficiency of RPE induction from pluripotent stem cells and removed the necessity for manually enriching patches of RPE from heterogeneous cultures [13, 14, 18] making the prospect of simultaneously generating multiple iPSC-derived RPE lines more practical and significantly expanding the scope of possible RPE research. However, many protocols still employ iPSCs generated with integrating vectors, complex aggregation steps or undefined reagents which contribute to variability in the final cell product and are unallowable or undesirable for clinical translation [32, 33]. The work we present here utilizes the recent RPE differentiation protocol from the Clegg group at UC Santa Barbara [14] that quickly and efficiently induced the differentiation conjunctiva-derived iPSCs into RPE regardless of donor and disease status. Without requiring protocol modifications for individual iPSC lines, the pattern and outcome of the RPE induction was similar between iPSC lines derived from the same or different donors and provided consistent yields of functional RPE for scalable cell expansion. The conjunctival iPSC-derived RPE exhibited characteristics that were structurally and functionally similar to RPE *in vivo*. Gene and protein expression matched that of RPE from adult retinas and the cells exhibited establishment of correct basolateral polarity, growth factor secretion and rod outer segment phagocytosis. Our successful implementation of a previously described protocol using multiple cell lines in a different laboratory setting is a significant validation of the utility of the method, highlighting the importance of design and testing of differentiation protocols using defined components and predicable process steps. To ensure that the process of generating conjunctiva-derived iPSC lines and their subsequent differentiation into RPE could also be entirely compliant with current good manufacturing practices necessary for clinical cell manufacturing, we demonstrated that the substitution of defined culture matrices did not alter the outcome of the RPE differentiation process.

## Conclusion

This work demonstrates an example of end-to-end manufacturing using defined culture and differentiation conditions suitable for the generation of a specific cell type from human iPSCs. The reprogramming and differentiation process described is simplified and can be implemented in a short timeframe with a relatively unspecialized skillset. The implementation and success of complex research projects requiring multiple lines of specialized cells derived from iPSCs or ESCs and the potential progression of these cells to widespread clinical use will rely entirely on the consistent availability of the functional cell product manufactured at an economical yield and cost. The successful use of human conjunctival cells in this work introduces an alternative patient cell source suitable for iPSC reprogramming using tissue from elderly human donors and with the differentiation process described, provides a standardized approach to generating RPE for both research and translation.

## Supporting information

S1 Fig Conjunctival cell yield from punch biopsies. **A** Diagram indicating positions for each 2mm conjunctival biopsy. **B** Phase image of biopsy tissue in culture (*) showing conjunctival cells expanded from the explant (scale bar = 50μm) **C** Graph showing the number of conjunctival cells cultured from explanted biopsies from each region (mean from three individual donors +/- SD).
(TIF)

S2 Fig Characterization of iPSCs derived from human conjunctival cells. Characterization of individual conjunctiva derived iPSC lines includes immunohistochemistry (**A**), confirmation of the loss of exogenous RNA vectors (**B**), maintenance of normal karyotype (**C**), qRT-PCR analysis of pluripotent stem cell associated genes (relative to expression in H9 ESCs) (**D**), generation of a complex teratoma in immune-compromised mice (**E**).
(TIF)

S3 Fig Comparison of gene expression during expansion of RPE generated from two iPSC lines derived from the same donor. Comparison of qRT-PCR analysis of markers of RPE phenotype. RPE lines UMN AMD1-1D3 and UMN AMD1-2B6 were derived from the same donor and differentiated in to RPE using the defined, rapid induction protocol. RPE cells from each line were then maintained in culture over 5 passages and the gene expression of key markers of RPE identity were measured for each line in each passage.
(TIF)
