# Supplementary figures and images for: Generation of retinal pigmented epithelium from iPSCs derived from the conjunctiva of donors with and without age related macular degeneration
Source: PLoS One. 2017 Mar 10;12(3):e0173575. doi: 10.1371/journal.pone.0173575 (PMC5345835; doi:10.1371/journal.pone.0173575)

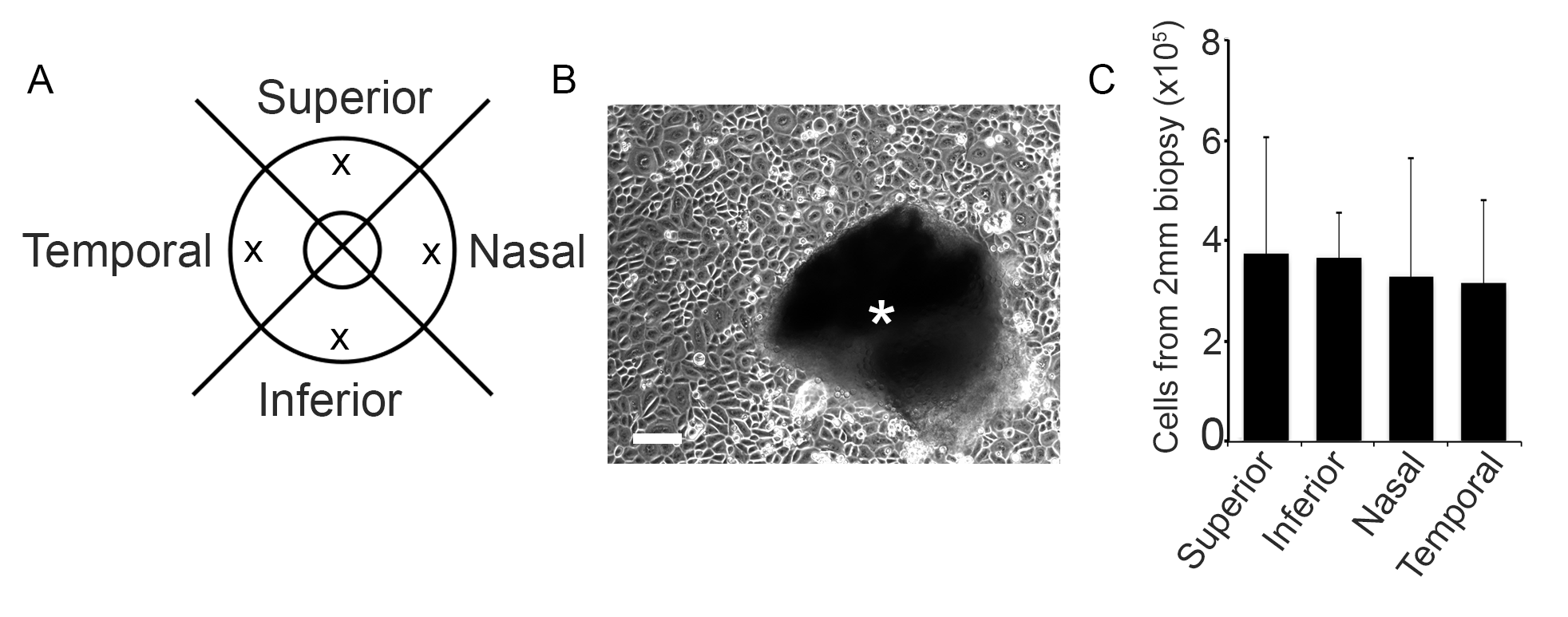

Supplement: S1 Fig — A Diagram indicating positions for each 2mm conjunctival biopsy. B Phase image of biopsy tissue in culture (*) showing conjunctival cells expanded from the explant (scale bar = 50μm) C Graph showing the number of conjunctival cells cultured from explanted biopsies from each region (mean from three individual donors +/- SD). (TIF) [file pone.0173575.s001.tif]

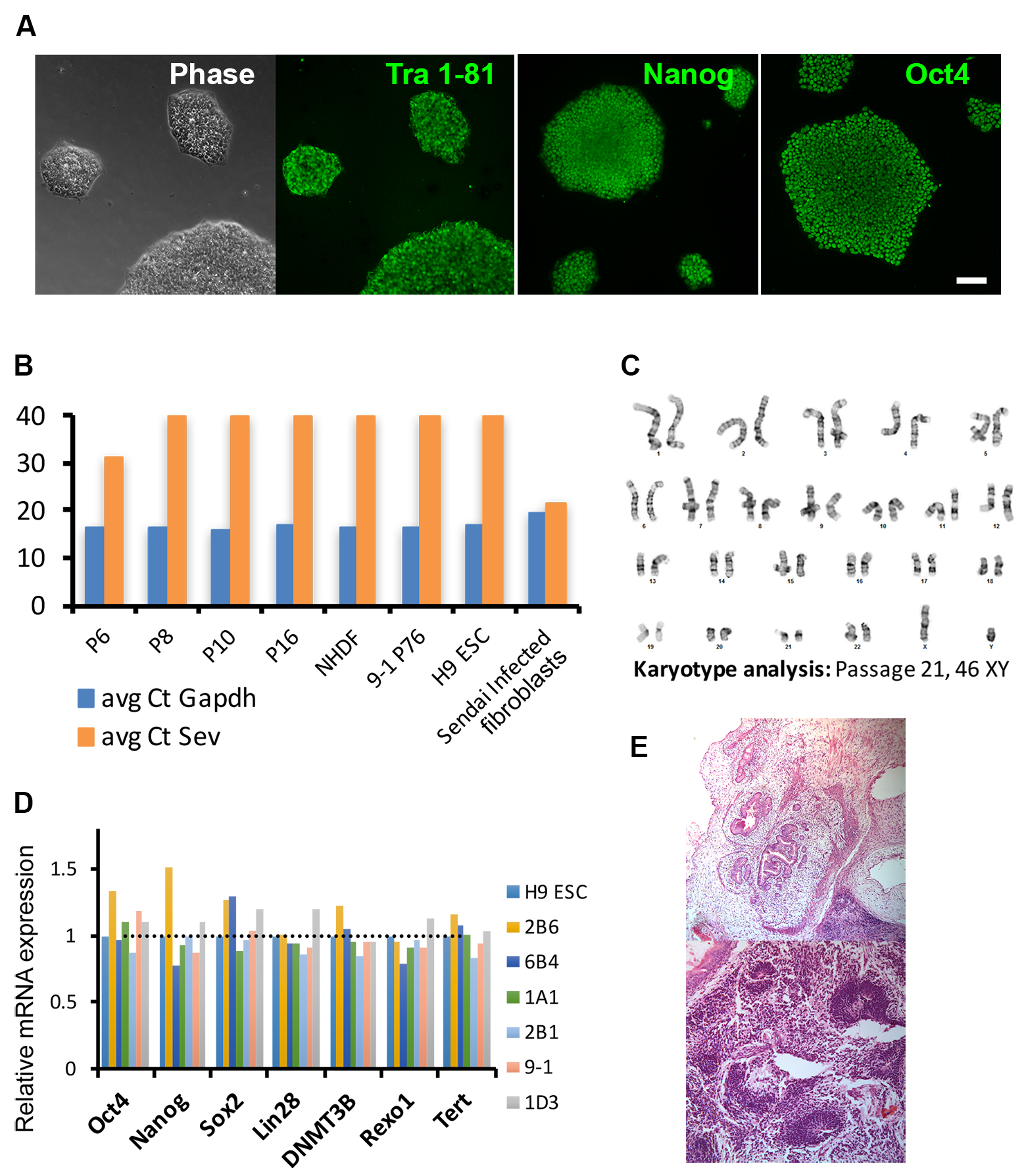

Supplement: S2 Fig — Characterization of individual conjunctiva derived iPSC lines includes immunohistochemistry (A), confirmation of the loss of exogenous RNA vectors (B), maintenance of normal karyotype (C), qRT-PCR analysis of pluripotent stem cell associated genes (relative to expression in H9 ESCs) (D), generation of a complex teratoma in immune-compromised mice (E). (TIF) [file pone.0173575.s002.tif]

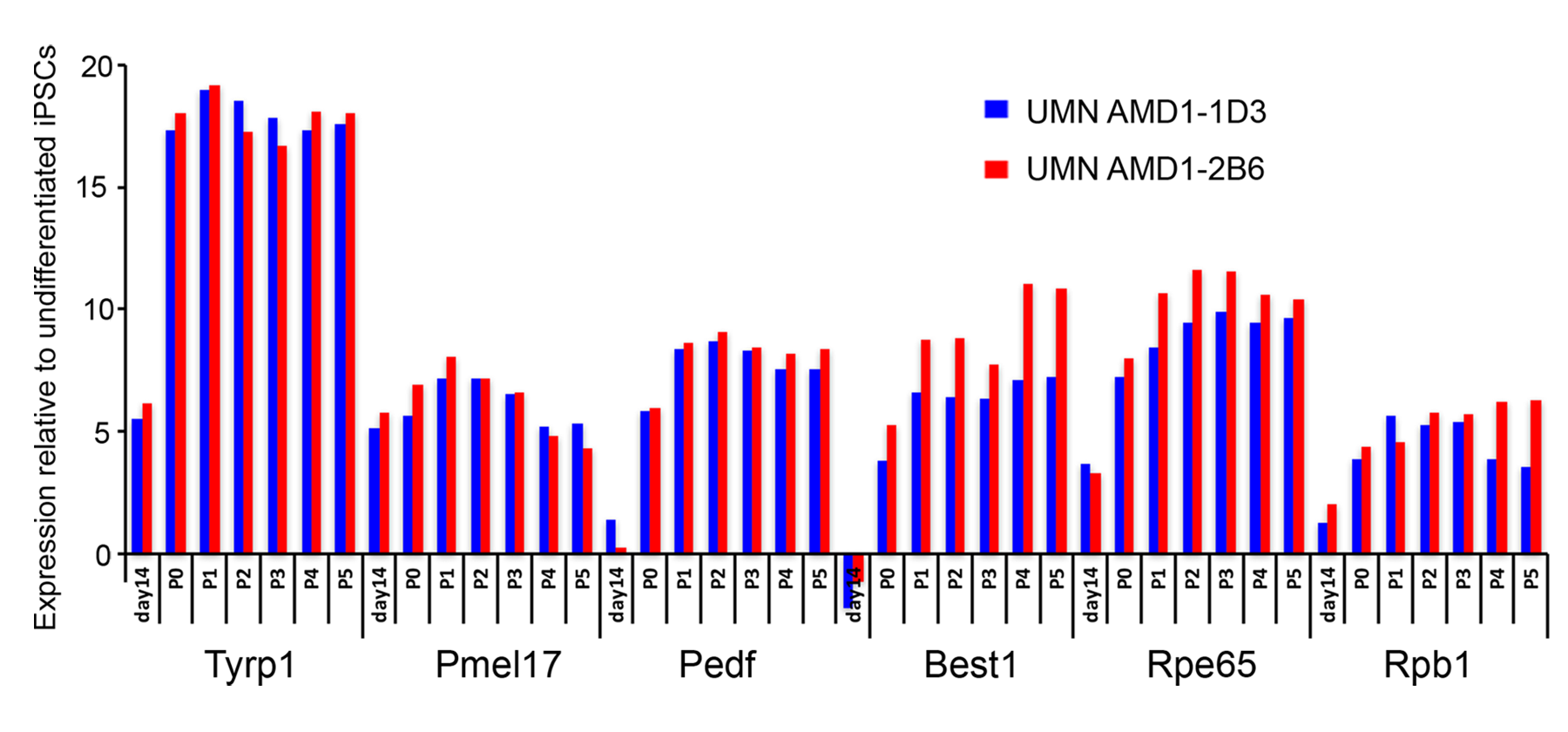

Supplement: S3 Fig — Comparison of qRT-PCR analysis of markers of RPE phenotype. RPE lines UMN AMD1-1D3 and UMN AMD1-2B6 were derived from the same donor and differentiated in to RPE using the defined, rapid induction protocol. RPE cells from each line were then maintained in culture over 5 passages and the gene expression of key markers of RPE identity were measured for each line in each passage. (TIF) [file pone.0173575.s003.tif]
